# A heterozygous splicing variant IVS9-7A > T in intron 9 of the *MAPT* gene in a patient with right-temporal variant frontotemporal dementia with atypical 4 repeat tauopathy

**DOI:** 10.1186/s40478-023-01629-3

**Published:** 2023-08-10

**Authors:** Kohji Mori, Kazue Shigenobu, Goichi Beck, Ryota Uozumi, Yuto Satake, Maki Suzuki, Shizuko Kondo, Shiho Gotoh, Yuki Yonenobu, Makiko Kawai, Yuki Suzuki, Yuko Saito, Eiichi Morii, Masato Hasegawa, Hideki Mochizuki, Shigeo Murayama, Manabu Ikeda

**Affiliations:** 1 Department of Psychiatry, Graduate School of Medicine, Osaka University, Yamadaoka 2-2, Suita, Osaka, Japan; 2 Department of Psychiatry, Asakayama General Hospital, Sakai, Japan; 3 Department of Behavioral Neurology and Neuropsychiatry, United Graduate School of Child Development, Osaka University, Suita, Japan; 4 Department of Neurology, Graduate School of Medicine, Osaka University, Suita, Japan; 5 Department of Pathology, Graduate School of Medicine, Osaka University, Suita, Japan; 6 Department of Psychiatry, Kansai Rosai Hospital, Amagasaki, Japan; 7 Brain Bank for Aging Research (Neuropathology), Tokyo Metropolitan Institute of Geriatrics and Gerontology, Tokyo, Japan; 8 Dementia Research Project, Tokyo Metropolitan Institute of Medical Science, Tokyo, Japan; 9 Brain Bank for Neurodevelopmental, Neurological and Psychiatric Disorders, Molecular Research Center for Children’s Mental Development, United Graduate School of Child Development, Osaka University, Suita, Japan

**Keywords:** Right-temporal variant frontotemporal dementia, MAPT, Behavioral variant frontotemporal dementia, Frontotemporal lobar degeneration, Intron, Splicing

## Abstract

Right temporal variant frontotemporal dementia, also called right-predominant semantic dementia, often has an unclear position within the framework of the updated diagnostic criteria for behavioral variant frontotemporal dementia or primary progressive aphasia. Recent studies have suggested that this population may be clinically, neuropathologically, and genetically distinct from those with behavioral variant frontotemporal dementia or left-predominant typical semantic variant primary progressive aphasia. Here we describe a Japanese case of right temporal variant frontotemporal dementia with novel heterozygous *MAPT* mutation Adenine to Thymidine in intervening sequence (IVS) 9 at position -7 from 3ʹ splicing site of intron 9/exon 10 boundary (*MAPT* IVS9-7A > T). Postmortem neuropathological analysis revealed a predominant accumulation of 4 repeat tau, especially in the temporal lobe, amygdala, and substantia nigra, but lacked astrocytic plaques or tufted astrocytes. Immunoelectron microscopy of the tau filaments extracted from the brain revealed a ribbon-like structure. Moreover, a cellular *MAPT* splicing assay confirmed that this novel variant promoted the inclusion of exon 10, resulting in the predominant production of 4 repeat tau. These data strongly suggest that the *MAPT* IVS9-7 A > T variant found in our case is a novel mutation that stimulates the inclusion of exon 10 through alternative splicing of *MAPT* transcript and causes predominant 4 repeat tauopathy which clinically presents as right temporal variant frontotemporal dementia.

## Introduction

Frontotemporal dementia (FTD) is a neurodegenerative disorder that predominantly affects the frontal and temporal lobes. Clinically, FTD encompasses three prototypical clinical syndromes: behavioral variant (bv)FTD, semantic dementia (SD), and progressive non-fluent aphasia [1–3]. Anterior temporal lobe atrophy in SD typically shows asymmetry, with episodic memory loss, behavioral disturbances, and prosopagnosia prominent in right-predominant SD, whereas word-finding difficulties and impaired comprehension are more salient in left-predominant SD [4–8]. Since the update of the diagnostic criteria in 2011, SD [1] has been placed within the framework of primary progressive aphasia (PPA) as semantic variant (sv)PPA [9]. As the initial diagnostic step of PPA excludes cases with “prominent, initial behavioral disturbance”; however, this change has led to relative ignorance of patients with prominent right anterior temporal lobe atrophy with early behavioral symptoms. To circumvent this issue, the concept of the right temporal variant (rtv)FTD [7, 10, 11] has recently been demarcated [12]. SD is genetically sporadic [13–15], with 72–83% of svPPA having FTLD-TDP type C pathology [16, 17], while genetic mutations have frequently been found in 33–35% of rtvFTD cases, and their neuropathology is diverse [11, 18, 19].

Genetic mutations in *MAPT* that cause FTD spectrum disorders are often found within exon 10 or in the stem-loop structure of the exon 10/intron 10 boundary. In contrast, only one disease-causing mutation has been reported in intron 9 of the *MAPT* gene [20]. Here, we report a case with atypical 4 repeat tauopathy. A novel *MAPT* variant in intron 9 found in this case stimulated exon 10 inclusion during splicing, thus causing the disease.

## Clinical description

**Fig. 1:**
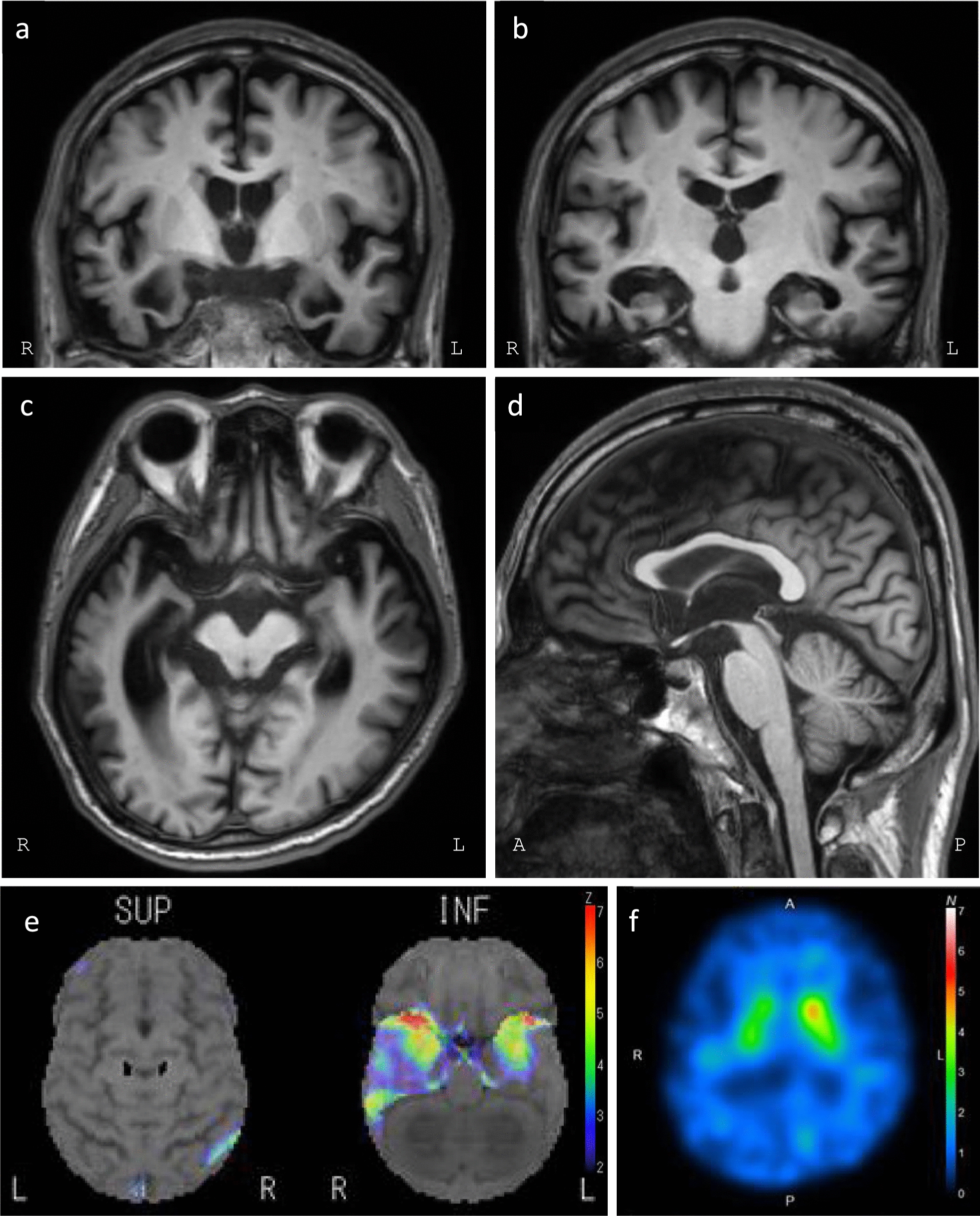
Clinical Images. **a**–**d** T1-weighted MR images at age 59. **a** Coronal image showing visible amygdala. Severe bilateral amygdala atrophy, predominantly on the right side, can be observed. White matter signal intensities in the left temporal lobe and right superior temporal gyrus are preserved, while those intensities in the right middle and inferior temporal gyri are attenuated. The third ventricle is enlarged. **b** Coronal image showing the hippocampal body. The hippocampus is mildly atrophic but relatively spared. **c** Axial image displaying predominantly enlarged right inferior horn of the lateral ventricle. Mild midbrain atrophy is noticeable. **d** Sagittal image revealing mild atrophy in the midbrain tegmentum area. **e** 3D-SSP image of ^123^I-IMP-SPECT demonstrates predominant right medial temporal hypoperfusion at age 57. **f** 123I-Ioflupane-SPECT reveals reduced binding to the striatal dopamine transporter, predominantly on the right side, at age 57

A right-handed 51-year-old man, who was an employee of a company, became more fastidious than before. When he was 52 years old, bizarre behaviors emerged, such as hoarding toilet paper and gum syrup and talking to his middle and high school sons as if they were toddlers. Difficulties in word comprehension were occasionally observed. At the age of 54, amnesia appeared, including forgetting yesterday’s events. He also developed a food preference for sweetness and a strictly fixed daily rhythm that looked like a timetable, such as going to a certain restaurant on the same day of the week at the same time of the day. At the age of 55, repetitive and obsessive checks of the power plug at home appeared. Disinhibitions such as spitting and loud monologues at work also worsened, and he was diagnosed with bipolar disorder at a nearby psychiatrist’s office. When he was 56, he was defrauded by an online scam worth over JPY 50 million. At the age of 57 years, he was admitted to a local psychiatric hospital for three months and discharged with multiple psychotropic medications. He began speaking strangely with prolonged endings. When his attending psychiatrist reduced his psychotropic medication, he began to lose control, talking late at night to his son, who was scheduled to take a college entrance exam. Therefore, he was referred to the psychiatry clinic of Osaka University Hospital, where an FTD-spectrum disorder was suspected during the initial examination. He was admitted to the psychiatric ward for a thorough examination. His wife said that his deceased, estranged father probably had young-onset dementia, although further family history details were unavailable. During the examination, the patient was highly distracted but cooperative. He lacked insight into his disease and had no sense of its seriousness. He exhibited agitation, indifference, disinhibition, abnormal motor behavior, disturbed sleep, and changes in eating behavior, which were scored using the neuropsychiatric inventory. His evaluation of the Stereotypy Rating Inventory [21] provided points for eating behavior, roaming, speaking, and movements. Neurological examination revealed mild rigidity and bradykinesia in both upper extremities (no difference between the right and left extremities). A tendency to disuse the left hand and an imitation behavior were observed. Neuropsychological batteries were as follows: Mini-Mental State Examination, 22/30 (orientation -2, serial sevens -2, three-stage command -1, delayed recall -3); Frontal Assessment Battery 14/18, Addenbrooke’s Cognitive Examination-Revised (ACE-R) [22], 55/100 (attention and orientation, 14/18; memory, 7/26; fluency, 10/26; language, 14/26; visuospatial, 10/16); and the aphasia quotient of the Western Aphasia Battery, 92.1, revealing a memory deficit with relatively spared attention and language. He could remember daily episodes with ward staff, suggesting his memory impairments were not severe. He showed impairment in recognizing famous landscapes (examined with our original quantitative test of famous landscape) or famous figures (assessed using the Visual Perception Test for Agnosia Famous Faces Test version 2 (VPTA-FFT ver 2) [23]), and he could not point to them even when instructed to do so; therefore, he was found to have some degree of semantic memory impairment. Clinically, the patient’s prosopagnosia was mild because he was able to recognize ward staff members’ faces after many encounters. He scored 90/200 on the picture-naming task and 148/200 on the auditory word comprehension task as assessed by the Test of Lexical Processing in Aphasia (TLPA) [24], suggesting mild impairments in semantic memory. Surface dyslexia was also observed in the reading irregular words subtest of the ACE-R, suggesting an impairment in the semantic memory of language. However, the object knowledge qualitatively assessed during the naming test of ACE-R was not clearly impaired. His magnetic resonance imaging (MRI) scan (representative images at age 59 years are shown in Fig. 1a–d) revealed marked atrophy of the right-dominant medial temporal lobe, including the amygdala, extending to the temporal pole. Hippocampal atrophy was relatively mild compared to severe atrophy in the amygdala. Caudate was preserved. In addition, there was severe atrophy in the basal part of the right temporal lobe. No obvious atrophy was observed in the parietal lobe or cerebellum; however, mild atrophy was observed in the midbrain tegmentum (Fig. 1c, d). The 3D-SSP analysis of ^123^I-IMP-SPECT showed reduced cerebral blood flow, predominantly from the right temporal pole to the mediobasal temporal lobe (Fig. 1e). ^123^I-Ioflupane-SPECT (DaT Scan™) at age 57 revealed right-predominantly reduced standard binding ratio (SBR R = 3.11, L = 5.38, Asymmetry Index 53.3%) (Fig. 1f). Electroencephalogram study revealed 8–9 Hz of basic rhythms with amplitude of 20–30 μV. No evident epileptiform discharges were observed. Blood tests revealed no remarkable findings. Cerebrospinal fluid examination revealed a cell count of 0/μl, total protein 46 mg/dl, and the amyloid beta 42/40 ratio was 0.084 (institutional cut off < 0.0705), suggesting that amyloid pathology is unlikely to be present; however, CSF phosphorylated (p)-tau181 was 54 pg/ml, which is slightly above the cut off (cut off > 50). The total CSF tau level was 338 pg/ml.

Based on these findings, the patient was diagnosed with right-predominant semantic dementia (rt-SD), also known as rtvFTD. Because the terms rt-SD and rtvFTD are often used to describe similar cases, we used the term rtvFTD in this study. Clinically, the patient met the criteria for behavioral variants of frontotemporal dementia (bvFTD) [2]. Given that it was already difficult for him to live at home because of pronounced behavioral symptoms, he was transferred to another psychiatric hospital for long-term treatment and care. After transfer, left-predominant muscle rigidity gradually appeared. No obvious abnormalities in eye movements, side-to-side differences in tendon reflexes, or pathological reflexes suggestive of pyramidal tract degeneration were observed at this time point.

At 59 years of age, the patient was transferred to the psychiatric ward of the university hospital for further examination. F^18^-Florbetapir Amyloid PET was negative, and Alzheimer’s disease was ruled out. The second round of neuropsychological batteries confirmed progressive deterioration within one year. In addition to executive dysfunction and personality changes, his trunk was leaning to the left, muscle stiffness and myoclonus appeared predominantly on the left side, and the clinical research criteria for probable sporadic corticobasal degeneration (based on frontal-behavioral spatial syndrome) [25] were also met. After examination, the patient returned to the psychiatric hospital. The patient’s spontaneity progressively declined. The patient developed food regurgitation and aspiration pneumonia, with repeated remission relapses. At the age of 60 years, his food intake gradually decreased, and muscle stiffness progressed, making it difficult for him to walk. However, he was able to eat orally, and he could name the side dishes of daily hospital meals until just before his death. The patient eventually died of sepsis.

## Neuropathology and biochemical analysis

**Fig. 2:**
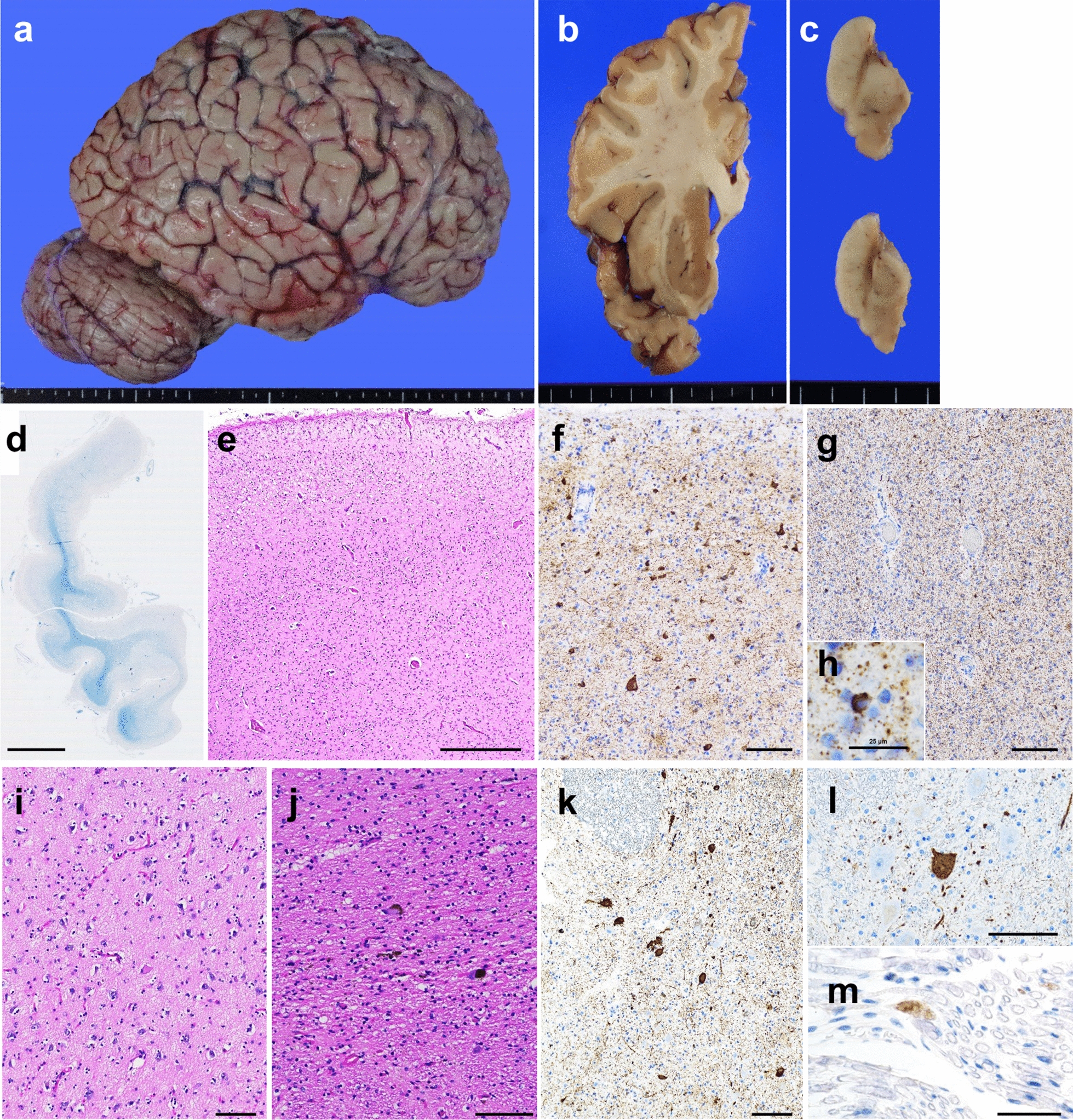
Neuropathological findings. **a**–**c** Macroscopic findings of the brain. **a** Severe atrophic changes in the temporal lobe are visible in the convex external view of the cerebrum. **b** Sections reveal atrophy of the temporal cortex. **c** The substantia nigra in the midbrain appears brownish at each level. **d** Semi-macroscopic findings of the temporal pole show severe atrophic changes and myelin pallor. **e**–**m** Microscopic findings of the temporal pole (**e**–**h**), amygdala (**i**), substantia nigra (**j**), locus coeruleus (**k**), cervical anterior horn (**l**), and cervical anterior root (**m**). Severe neuronal loss with gliosis is visible in the temporal pole (**e**), amygdala (**i**), and substantia nigra (**j**). Prominent accumulation of p-tau is observed in the cortex (**f**) as well as the white matter (**g**) of the temporal pole. A glial cytoplasmic inclusion with high magnification is shown in (**h**). Neuronal/glial inclusions and threads positive for p-tau are frequently visible in the locus coeruleus (**k**). Anterior horn cells (**l**) and anterior roots (**m**) in the cervical cord show positive staining for p-tau. KB staining (**d**); HE staining (**e**, **i**, **j**); immunohistochemistry for p-tau (**f**–**h**, **k**–**m**). Scale bars: 5 mm in (**d**), 500 µm in (**e**), and 100 µm in (**f**, **g**, **i**–**l**), 50 µm in (**m**), and 25 µm in (**h**)

The brain weighed 1132 g before fixation and macroscopic observation revealed severe atrophic changes in the bilateral temporal lobes and mild atrophic changes in the bilateral frontal lobes (Fig. 2a, b). In the sections, the substantia nigra was brownish (Fig. 2c), and depigmentation of the locus coeruleus was visible in the brainstem.

**Fig. 3:**
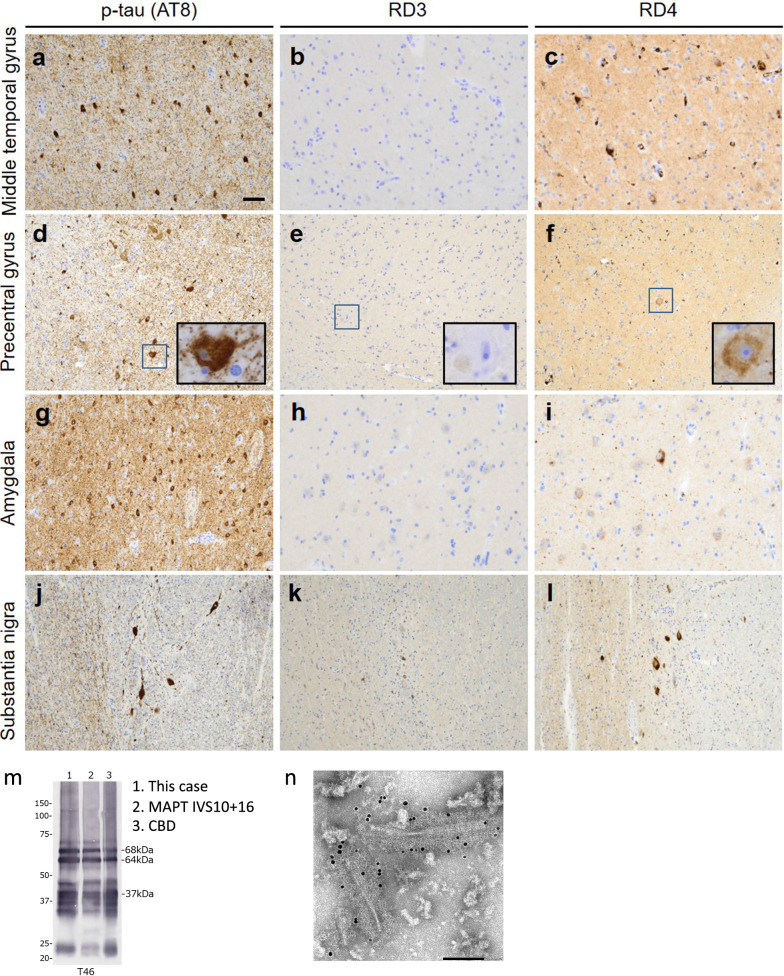
Tau pathology of the present case. **a**, **d**, **g**, **j** Numerous p-tau-positive neuronal and glial inclusions, as well as threads, are visible in the middle temporal gyrus (**a**), precentral gyrus (**d**), amygdala (**g**), and substantia nigra (**j**). These inclusions and threads are positive for RD4 (**c**, **f**, **i**, **l**) but negative for RD3 (**b**, **e**, **h**, **k**). **d**–**f** The insets, which show Betz cells, are high magnification of squares in each panel. Betz cells are positive for p-tau and RD4 but negative for RD3. Immunohistochemistry of p-tau (**a**, **d**, **g**, **j**), RD3 (**b**, **e**, **h**, **k**), and RD4 (**c**, **f**, **i**, **l**). Scale bar in (**a**) represents 200 µm (**a**, **d**–**g**) and 100 µm (**b**, **c**, **h**–**l**). **m** Immunoblot analyses of sarkosyl-insoluble fractions prepared from the brain of this patient and tauopathy patient references. Sarkosyl-insoluble full-length tau (64 kDa and 68 kDa) and C-terminal fragments (33 kDa and 37 kDa) were detected using the T46 antibody (residues 404–441). **n** Immunoelectron microscopy of sarkosyl-insoluble fractions extracted from the brain. An electron micrograph shows twisted filaments that are positive for the AT8 antibody

Microscopic examination revealed severe neuronal loss and gliosis in the temporal pole (Fig. 2d, e), superior/middle/inferior temporal gyrus, amygdala (Fig. 2i), substantia nigra (Fig. 2j), and locus coeruleus. Mild-to-moderate neuronal loss with gliosis was also visible in several brain regions, including the superior/middle/inferior frontal gyrus, hippocampus, anterior cingulate gyrus, subthalamic nucleus, and red nucleus. Immunohistochemistry for p-tau revealed numerous neuronal intracytoplasmic inclusions, glial inclusions, and threads in the temporal pole (Fig. 2f–h), superior/middle/inferior temporal gyrus (Fig. 3a), precentral gyrus (Fig. 3d), parietal cortex, amygdala (Fig. 3g), hippocampus, anterior cingulate gyrus, transentorhinal cortex, substantia nigra (Fig. 3j), and locus coeruleus (Fig. 2k). Betz cells were strongly positive for p-tau (Fig. 3d). Mild-to-moderate amounts of p-tau-positive neuronal/glial inclusions were visible in several brain areas, including the superior/middle/inferior frontal gyrus, precuneus, occipital cortex, putamen, globus pallidus, subthalamic nucleus, pontine nucleus, reticular formation of the pons, inferior olivary nucleus, dentate nucleus of the cerebellum, anterior horn of the spinal cord (Fig. 2l), and anterior roots (Fig. 2m). These inclusions were positive for RD4 (Fig. 3c, f, i, l) but negative for RD3 (Fig. 3b, e, h, k) in all examined areas. No astrocytic plaques or tufted astrocytes are observed. No immunohistochemical staining was detected for amyloid-β (Thal phase 0) [26]. No Lewy or TDP-43 pathology was observed.

Western blotting of sarkosyl-insoluble tau from the frozen brain, in this case, showed a banding pattern similar to that of the *MAPT* IVS10 + 16 mutation and corticobasal degeneration (CBD) cases (Fig. 3m) [27]. Immunoelectron microscopy of the sarkosyl-insoluble fraction revealed a ribbon-like morphology. Thin fibers corresponding to the protofilaments were also visible. (Fig. 3n).

## Molecular genetics and MAPT splicing assay

**Fig. 4:**
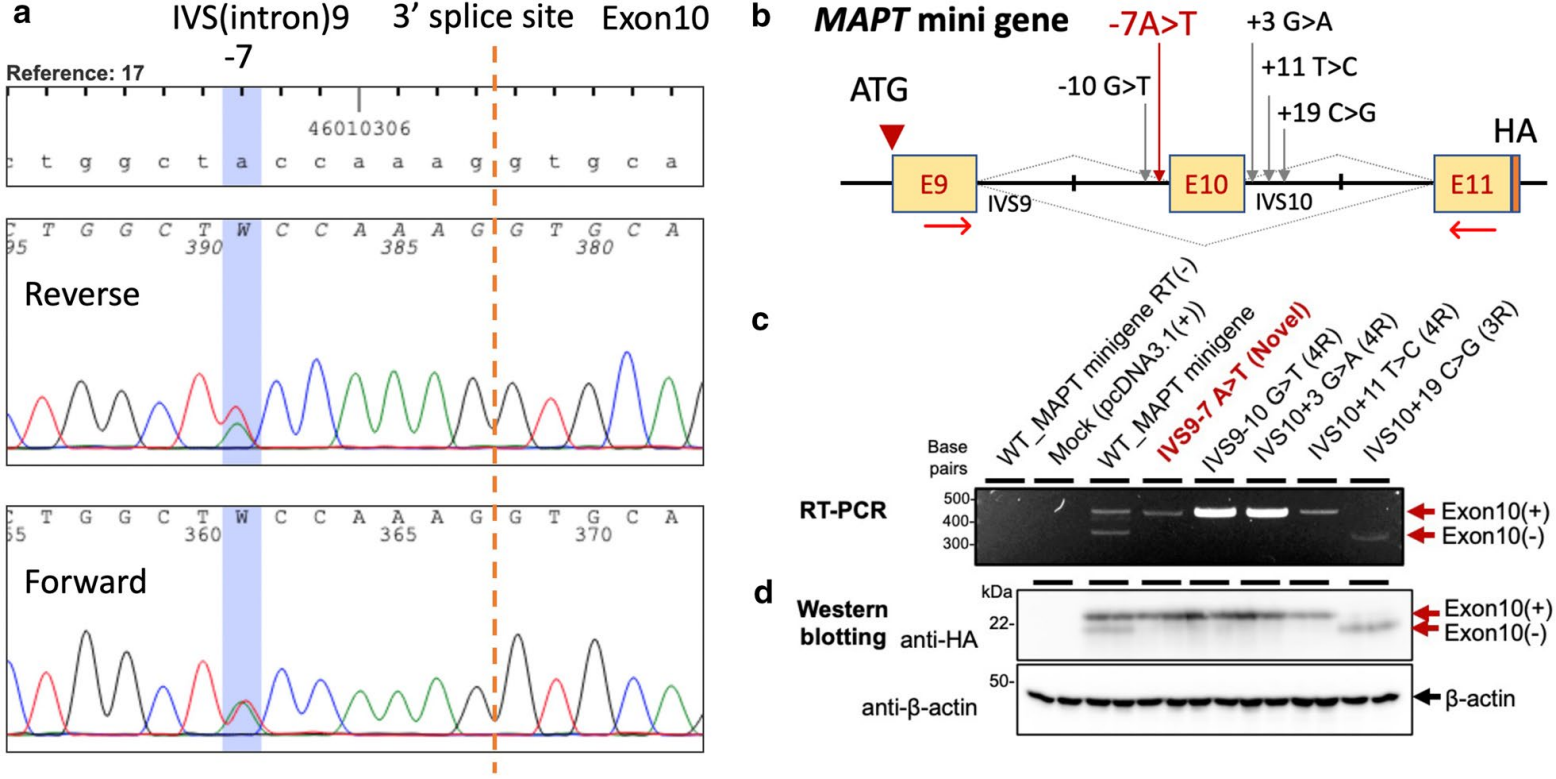
Genetic analysis and *MAPT* splicing assay. **a** Sanger sequencing of *MAPT* intron 9/exon10 boundary, displaying a reference sequence (top) and two consistent electropherograms (reverse and forward directions). **b** Schematic diagram depicting the design of *MAPT* mini gene construct with or without disease-causing mutation/variant. **c** Reverse Transcription Polymerase Chain Reaction (RT-PCR) of *MAPT* minigene transcripts (3 independent experiments). Complementary DNAs were collected from HeLa cells transfected with each plasmid. **d** Western blotting analysis of transfected HeLa cells in duplicate probed with anti-HA tag antibody (two independent experiments). Bands corresponding to 4 repeat tau (Exon 10 (+)) and 3 repeat tau (Exon 10 (−)) were observed. Anti-β-actin antibody was used as protein loading controls. RT(−): no reverse transcription. Mock indicates backbone plasmid vector lacking *MAPT*-related sequence. Exon 10 (+) and (−) indicate bands corresponding to exon 10 inclusion and exclusion

Sanger sequencing was performed on all exons and flanking regions of the *MAPT* gene using blood-derived DNA obtained antemortem. We found no known pathological variants in any of the exons, including exon 10 and the loop domain spanning the exon 10 and intron 10 boundary of the *MAPT* gene. Instead, we noticed a novel variant in IVS9-7 A > T (c.937-7A > T of RefSeq NM_005910.6 (= c.823-7A > T of NM_005910.5)) in an allele. This variant locates at -7 position of 3ʹ splice site of *MAPT* exon 10 according to a common alternative exon numbering convention (Fig. 4a). No common SNP was reported in dbSNP, and no variant was reported in gnomAD v2.1.1 in this locus (database search on Jan 25th, 2023). A previous biochemical study suggested this variant locate within a suboptimal polypyrimidine tract region (IVS9-21 to -4), which locates between the branch point sequences (IVS9-32 to -26, IVS9-28 to -22) and the 3ʹ splice site of intron 9/exon 10 boundary [28]. In their study, the introduction of two sets of tripartite purine (A, G) to pyrimidine (T, C) mutations ((IVS9-11G > T, -10G > T, -7A > T) or (IVS9-7A > T, -4A > T, -3A > T)) that increased the number of consecutive pyrimidines in their *MAPT* minigene enhanced the efficacy of exon 10 inclusion [28]. Interestingly the IVS9-7A > T mutation identified in our case was shared between two consecutive pyrimidine constructs [28].

The inclusion of exon 10 in RNA splicing results in the production of 4 repeat tau. To examine the effect of the novel IVS9-7 A > T variant on exon 10 splicing, several *MAPT* minigene constructs were generated (Fig. 4b, also see the Methods section). When the *MAPT* minigene with the reference (wild-type: WT) sequence was transfected into HeLa cells, two bands corresponding to exon 10 (+) and exon 10 (−) transcripts were detected by RT-PCR analysis (Fig. 4c, top panel, third lane from the left). In the presence of the IVS9-7A > T variant on the *MAPT* minigene (Fig. 4b), the predominant expression of exon 10 containing transcript was observed (Fig. 4c, top panel, fourth lane from the left). To further validate the reliability of our splicing assay, each of the previously reported *MAPT* non-cording mutations was introduced into the minigene constructs. The expression of *MAPT* minigenes with 4-repeat tau predominant FTD-causing mutations (IVS9-10G > T [20, 29], IVS10 + 3G > A, [30] and IVS10 + 11T > C [31, 32]) confirmed the preferential expression of exon 10 containing transcripts (Fig. 4b, c). Conversely, the expression of a *MAPT* minigene with a 3-repeat tau predominant FTD-causing mutation (IVS10 + 19C > G [33]) resulted in the selective detection of exon 10 excluded transcripts (Fig. 4b, c). These results at the transcript level were further confirmed by western blotting of transfected cell lysates (Fig. 4c).

Our analysis confirmed that *MAPT* IVS9-7A > T variant promotes the balance of *MAPT* splicing toward predominant 4 repeat tau expression. This is consistent with the neuropathological findings of observed exclusive 4 repeat tau accumulation. Therefore, we conclude that the *MAPT* IVS9-7A > T variant is causative for 4 repeat tauopathy and clinically presents as rtvFTD.

## Discussion

In our case, the prominent early behavioral symptoms could be attributed to severe atrophy of the right temporal lobe. Although harmonized clinical diagnostic criteria for rtvFTD have not yet been developed, prosopagnosia, memory deficits, getting lost and profound behavioral changes such as disinhibition and obsessive personality are the main clinical characteristics associated with rtvFTD [12, 34]. In typical SD cases, spontaneous speech often disappears or decreases significantly in later stages. However, it should be emphasized that in the present case, although the number of words was reduced, the patient was still able to call out items (contents of side dishes in the daily hospital meal) until the end of life. Although a formal examination was not possible, this suggests that a serious impairment of semantic memory was not present until the end of his life. While SD is genetically sporadic [13–15], genetic mutations in FTD-related genes were found in 33–35% of genetically screened rtvFTD cases [11, 18]. The case described herein is an additional example of rtvFTD with mutations in FTD-related genes. Neuropathologically, FTLD-TDP type C accounts for only 36% of rtvFTD cases and is more heterogeneous than left-predominant SD, with FTLD-type C accounting for 80% of cases [35].

As for the multilayered clinical diagnosis of CBD, the criteria for probable sporadic CBD were no longer met owing to identifying a *MAPT* mutation; however, the diagnosis of possible CBD is still on hold [25]. Our biochemical analysis of sarkosyl-insoluble filaments from frozen brains showed similar banding patterns and filament structures as the other four repeat tauopathies.

Mechanistically, FTD spectrum disorders caused by *MAPT* exon 10 splice mutations are frequently found at the Exon10/intron10 boundary. In contrast, disease-causing mutations in the intron9/exon10 boundary are rare. Before our report, the *MAPT* IVS9-10G > T variant described in two FTDP-17 families was the only pathogenic mutation identified in IVS9 of *MAPT* [20, 29]. In accordance with our case, the IVS9-10G > T mutation is also a purine-to-pyrimidine mutation, resulting in the accumulation of 4 repeat tau in the postmortem brain.

Although our variant could be classified as “likely pathogenic” (PS3 + PM2) according to the ACMG guideline for the interpretation of sequence variants [36], our results from splicing assay and neuropathological examination strongly suggest that this variant induces preferential inclusion of exon 10 of *MAPT*, a well-established mechanism leading to 4 repeat tauopathy. Therefore, we conclude that the *MAPT* IVS9-7 A > T variant found in our case is a novel mutation that causes predominant 4 repeat tauopathy clinically presenting with rtvFTD. This case and previous reports highlight the importance of the polypyrimidine tract region in the 3ʹ part of intron 9 during the splicing regulation of the exon 10 inclusion of *MAPT*, as postulated more than 20 years ago [28].

## Experimental procedures

### Neuropathological examination

Autopsy was performed 4 h and 10 min after his death. The right hemisphere of the brain and the spinal cord were fixed in 10% buffered formalin for pathological assessment. The remaining brain was frozen for molecular and biochemical analyses. After macroscopic observation, appropriate areas were dissected and embedded in paraffin. Serial sections of 6 μm thickness were stained with hematoxylin, eosin, and luxol fast blue/cresyl violet (also known as Klüver-Barrera [KB] staining).

For immunohistochemistry, the primary antibodies used were mouse monoclonal antibodies against phosphorylated tau (p-tau) (clone AT8; Fujirebio, Japan; 1:1000), 3-repeat isoform tau (RD3) (clone 8E6/C11, Merck, Darmstadt, Germany, 1:2000), 4-repeat isoform tau (RD4) (clone 1E1/A6, Merck, Darmstadt, Germany, 1:1000), amyloid-β (clone 12B2; IBL, Japan; 1:100), and phosphorylated α-synuclein (p-α-syn) (clone pSyn#64; FUJIFILM Wako Pure Chemical, Japan; 1:1000), and a rabbit polyclonal antibody against TDP-43 phosphorylated at serine residues 409 and 410 (S409/S410) (p-TDP-43) (Cat. No. 22309-1-AP; Protein Tech Group, Chicago, IL, USA; 1:3000). Protease K treatment (10 μg/ml for 30 min) and autoclave treatment (10 min) were performed before incubation with the anti-RD3 and anti-RD4 antibodies.

### Extraction of tau filaments

To biochemically detect the abnormal accumulation of p-tau, a sarkosyl-insoluble pellet was prepared from the frozen brain as described previously [27] using the following steps. Frozen tissue from the patient’s temporal pole was homogenized in 40-fold volumes (v/w) of extraction buffer containing 10 mmol/L Tris–HCl (pH 7.5), 0.8 mol/L NaCl, 10% sucrose, and 1 mmol/L ethyleneglycoltetraacetic acid. The homogenates were treated with 2% sarkosyl and incubated for 30 min at 37 °C. Following a 10 min centrifugation at 27,000×*g* for 10 min at room temperature, the supernatants were ultracentrifuged at 166,000×*g* for 20 min at room temperature. The resulting pellets (insoluble fractions) were subjected to immunoblot analysis. After sodium dodecyl sulfate–polyacrylamide gel electrophoresis, the proteins were transferred onto a polyvinylidene fluoride membrane and detected using an anti-p-tau antibody (mouse monoclonal, clone T46; Thermo Fisher Scientific; 1:1000).

### Sanger sequence

Sanger sequencing was performed for all exons and flanking regions of MAPT using blood-derived DNA.

### Cellular splicing assays

A *MAPT* minigene plasmid consisting of exon 9 to exon 11 of a reference gene sequence (NG_007398.2), including the first and last 500 base pairs of introns 9 and 10, but excluding the middle region of each of introns 9 and 10, fused with an N-terminal translation initiation codon and C-terminal HA tag, was synthesized and cloned into the pcDNA3.1 (+) vector at KpnI/NotI sites (Fig. 4b). Corresponding disease-causing point mutations were introduced (GenScript). Plasmid sequences were verified by Sanger sequencing. The plasmids were transfected into HeLa cells using Lipofectamine LTX (Invitrogen). Following overnight culture, total RNA was extracted and reverse-transcribed into cDNA using MMLV reverse transcriptase and oligo dT. PCR analysis for spliced MAPT with primers Fw: 5' TGAACCTCCAAAATCAGGGGATCGC 3ʹ Rev:5ʹ CGCCGCTGGTTTATGATGGATGTTGCC 3ʹ was performed. Transfected cells were lysed with RIPA buffer, electrophoresed, and transferred onto a PVDF membrane for protein analysis. The immunoblot signals were obtained using anti-HA (3F10; Roche) or anti-β-actin (AC-74; Sigma-Aldrich) antibodies.

## Data Availability

Data are available from the corresponding author upon request.

## Abbreviations

bvFTD: Behavioral variant frontotemporal dementia
CBD: Corticobasal degeneration
DNA: Deoxyribonucleic acid
FTD: Frontotemporal dementia
FTLD: Frontotemporal lobar degeneration
IMP: N-isopropyl[123I]-p-iodoamphetamine
IVS: Intervening sequence
MAPT: Microtuble associated protein tau
MRI: Magnetic resonance imaging
PET: Positron emission tomography
PPA: Primary progressive aphasia
p-tau: Phosphorylated tau
RNA: Ribonucleic acid
RT-PCR: Reverse transcription polymerase chain reaction
rtvFTD: Right temporal variant frontotemporal dementia
rt-SD: Right-predominant semantic dementia
SD: Sementic dementia
SNP: Single-nucleotide polymorphism
SPECT: Single-photon emission computed tomography
svPPA: Semantic variant primary progressive aphasia
TDP: Transactive response DNA binding protein 43 kDa
